# The Epimmunity Theory: The Single Cell Defenses against Infectious and Genetic Diseases

**DOI:** 10.3389/fimmu.2017.00694

**Published:** 2017-06-13

**Authors:** Sameer A. Barghouthi

**Affiliations:** ^1^Faculty of Health Professions, Department of Medical Laboratory Sciences, Al-Quds University, Jerusalem, Palestine

**Keywords:** erythrocytes, CD71 receptor, malaria, enucleation, cancer, extravasation, metastasis, reticulocyte

## Abstract

Single cell defense against diseases defines “epimmunity.” Epimmunity is complementary to the immune system and can neither be substituted by innate nor by acquired immunity. Epimmunity, the proposed new branch of immunity, is further explored and analyzed for enucleated mature mammalian erythrocytes and nucleated erythrocytes of non-mammalian vertebrates leading to the development of “The Epimmunity Theory.” Enucleation of mammalian erythroblast and inactivation of nuclei in erythrocytes of non-mammalian vertebrates are major contributors to the collective immunity: epimmunity, innate, and acquired. The fact that diseases of mature erythrocytes (MEs) are rare supports the notion that a single cell can resist microbial and genetic diseases; MEs are refractory to malaria and cancer. Nucleated cells, such as B-cells, T-cells, hepatocytes, and cell developmental stages are susceptible to genetic and specific microbial diseases depending on their nuclear activities and the receptors they express; such cells show lower epimmunity relative to MEs. Epimmunity is important as a disease insulator that prevents the spread of diseases from an infected tissue to the majority of other tissues. Breakdown of epimmunity may lead to disease development.

## Introduction

The immune system of vertebrates indirectly protects tissues and cells against diseases. However, when cells are in direct contact with pathogens, the immune system is helpless and cannot prevent colonization, infection, invasion, or mutation of target cells. Yet, single cells may resist or evade colonization, infection, invasion, and can repair DNA breaks and mutations as well. Although DNA repair mechanisms have been reported in prokaryotes and eukaryotes, there have been no reports on cell evasion or defense against diseases. Hence, this report on single cell defense against disease is presented as “The Epimmunity Theory.”

### Hypothesis and the “Epimmunity Theory”

#### Hypothesis

Cell susceptibility to diseases is directly related to its nuclear activity, whereas cell epimmunity (single cell defense against disease) is inversely related to its nuclear activity.

Epimmunity describes single cell defense against genetic and infectious diseases. These defenses include nuclear activities, metabolic behavior, structural components, and other cellular activities including intracellular immunity. This report will focus on the role of enucleation and nuclear inactivation in the epimmunity of mature erythrocytes (MEs) of vertebrates against genetic and infectious diseases; the mature enucleated mammalian erythroblast (the erythrocyte; mME) and the nucleated non-mammalian mature erythrocytes (nME). It should be emphasized here that individual cells have differential abilities to defend themselves and may utilize different strategies in doing so; these strategies are dictated by cell genetic response to its microenvironment; Section “Viral Attachment, Agglutination, Hemadsorption, and Cell Fusion” (1).

Due to the critical biological function of erythrocytes and their large numbers, vertebrates will die shortly if their erythrocytes were to malfunction due to an infectious or genetic disease; hence they must be epimmune and refractory to diseases. Achieving this task requires the termination or suppression of nuclear function as illustrated in Section “ME Have to be Refractory to Genetic Diseases” on genetic diseases and Section “Infectious Diseases” on parasitic, bacterial, viral, and fungal diseases. Termination of nuclear activity is achieved in mME by enucleation or by nuclear inactivation in nME, leading to maximum protection of ME against all diseases. On the other hand, microbes may exploit cellular weaknesses to mount successful infections.

According to the literature, there is no single disease that directly afflicts ME; diseases of erythrocytes including sickle cell anemia, thalassemia, genetic or viral pure-red-cell aplasia (PRCA), and malaria are known to originate in erythrocyte progenitor cells. Although, viruses may interact with erythrocytes, yet they are incapable of replicating inside erythrocytes (1–3).

Incomplete or partial epimmunity can be observed in all cells showing differential gene expression (i.e., different cell surface receptors). Cells expressing one receptor or more jeopardize their own epimmunity; they become susceptible to genetic and infectious diseases depending on their nuclear activities. Differential gene expression of tissues is a reflection of nuclear activity in each tissue, leading to differential cell surface structures and receptors. Tissue variations provide a barrier that confines an infectious disease to a particular tissue(s) preventing it from spreading to other tissues, i.e., quarantine a disease to that tissue. Host specificity, host range, and tissue tropism (4) of microbes are caused by variations among tissue receptors of an individual and by variations among similar tissues in different individuals especially in reference to cell-surface receptors. These terms are rooted in epimmunity. Partial epimmunity renders a cell susceptible to some infections, it is exemplified by tissue tropism of Hepatitis B virus which infects hepatocytes, yet it has not been reported to infect several other tissues such as pneumocytes, myocytes, monocytes, or neurons. Whereas Epstein–Barr virus is known to target B-cells but not hepatocytes.

Epimmunity is conferred on the population by individual differences caused by allelic gene variations (e.g., ABO blood groups), single nucleotide polymorphism (SNPs), gene imprinting, epigenetic gene regulation (5), and quantitative gene expression.

Other areas linked to epimmunity include immune tolerance, self-recognition, allograft, xenograft, and autoimmune diseases.

Here, the “epimmunity theory” will be illustrated by focusing on vertebrate MEs to show that epimmunity among other factors have forced enucleation of mammalian erythroblasts, nuclear inactivation in non-mammalian vertebrates, and possibly partial inactivation of genes and chromosomes of mature differentiated cells. Available literature strongly supports the epimmunity theory; no contradicting reports have been identified; only inaccurate reports were suspected (studies referring to peripheral red cells as a homogenous ME population and not taking different stages and ages of circulating red cells into consideration).

## Epimmunity of MEs

During hematopoiesis, cells progress from one developmental stage to the next until erythroblasts finally mature into erythrocytes. Each developmental stage expresses different genes and surface receptors that distinguish one stage from others. Different tissues of an individual and similar tissue from different individuals vary in their surface receptors rendering some of them susceptible or resistant to a given pathogen. Such variations suggest that a single disease cannot equally strike a large population.

On the other hand, if a multicellular organism expressed a given receptor by all cells, then most probably, all cells will be at risk of being infected by the same pathogen with an inevitable devastating outcome to the host. Due to ME large numbers, critical function, and circulation, it is imperative that they are refractory to both genetic and infectious diseases.

### MEs Have to Be Refractory to Genetic Diseases

Genetically active cells are susceptible to genetic diseases (mutations, chromosome aberration, and cancer). They are also prone to infections depending on the receptors they express; cellular receptors can be parasitized by a variety of viruses (6). The following hypothetical example illustrates the importance of enucleation of mammalian erythroblasts and the inactivation of nuclei of avian erythrocytes (7) (and other vertebrates) in evading genetic and infectious diseases. Hypothetically, if one particle of a lytic virus were to release 50 particles every hour after invading a single human ME, then all circulating MEs of an individual will be lysed and death will occur in few hours (less than 10 h post infection).

Thalassemia, sickle cell anemia, spherocytosis, PRCA, and erythroid and bone marrow cancers are major genetic diseases of progenitor cells of erythrocytes (8–10). However, none of these diseases is known to initiate in ME. Enucleation of mammalian erythroblasts has circumvented erythrocyte susceptibility to genetic disease; they are genetically epimmune. Accordingly, mature mammalian erythrocytes are protected against genetic diseases including cancer. On the other hand, non-mammalian vertebrates have Nucleated Mature Erythrocytes and they achieved a similar level of epimmunity to mME by adopting a different, yet efficient, strategy. The avian erythrocyte nucleus is genetically inactive, i.e., dormant, or metabolically inactive (11). All vertebrate erythrocyte nuclei become condensed and transcriptionally inactive (12). Avian erythrocyte nucleus is retained in an inactive transcriptional state during terminal erythropoiesis; erythrocytes show no transcription, no DNA synthesis, and no protein synthesis including hemoglobin (13). Avian erythrocytes have active mitochondria (14) that are lost together with ribosomes and cellular RNA in aging nucleated erythrocytes (13, 15). In rainbow trout fish, erythrocytes experience many changes throughout their 4- to 6-month life span including loss of mitochondria, ability to mount heat shock response, and reduction in biosynthetic processes (7).

Nuclei of avian erythrocytes are unable to synthesize detectable RNA (including globin RNA) (16). Ninety percent of RNA is degraded within 8 days in the young circulating avian embryonic erythrocytes, degradation of RNA continues to near zero over the following 10 days (17), indicating the absence of RNA synthesis as well.

Dormant hen nuclei inserted into the cytoplasm of HeLa human cell line increase in size and transform from elliptic shape to become spherical (~3 μm diameter). Then they are induced to resume RNA and DNA synthesis (18).

Heterogeneous nuclear ribonucleic acid (hnRNA) metabolism in differentiating avian erythroid cells revealed the existence of stable hnRNA derivatives (19); the function of which was not clarified. Does it behave as an antisense RNA, an inhibitor of DNA replication, and/or transcription?

This question is raised again; avian erythrocytes synthesize messenger-like RNAs *via* endogenous polymerase II activity, these include polyadenylated species but contain no mRNA (16). Gene repression and chromatin condensation are accomplished by histone H5 and a 42 kDa-basic non-histone protein known as Mature Erythrocyte Nuclear Termination stage-specific protein (MENT) that accumulates in erythrocyte nuclei of adult chicken (20). However, *in vitro* LPS-stimulated rainbow and chicken erythrocytes were able to increase levels of Toll-like receptor transcription, supporting the conclusion that nMEs are transcriptionally active and can translate TLR mRNA [St Paul et al. (21) and others] which contradicts earlier reports (11–13, 16, 18, 20, 22, 23). These conflicting reports need to be sorted out and carefully analyzed to clarify this discrepancy.

In general, differentiated cells have partially active nuclei as indicated by the many tissue types of vertebrates (i.e., hepatocyte vs myocyte or neurons) rendering them partially susceptible to phenotypic mutation (i.e., with some exceptions, mutated genes that are inactive will have no consequences on the inert gene and the phenotype of that cell). Similarly, these cells will only engage microbes through their limited surface components which is subject to modification by mutation or interaction with intrinsic immune complexes or extrinsic drugs (cefotetan, ceftriaxone, and piperacillin) as in drug-induced immune hemolytic anemia (24, 25).

Epigenetics, histone code, gene imprinting, iRNA, anti-sense RNA, and their combination are likely to play important roles in achieving tight regulation of nuclear activities, arrest cell differentiation, cell division, and inactivate nuclei of nME.

Such nuclear inactivation renders MEs refractory to genetic diseases, similar to enucleated mammalian erythrocytes.

### Infectious Diseases

Claims of parasitic protozoan, bacterial, fungal, and viral diseases of ME are vague and unsubstantiated as revealed by examining some of these infectious diseases of “erythrocytes.”

During final stages of enucleation of mammalian erythroblast, certain receptors are depleted from reticulocytes by way of vesicle sorting and trafficking of proteins to pyrenocytes or reticulocytes (26, 27). Transferrin CD71 receptor is completely eliminated from reticulocytes by sorting to pyrenocytes, unlike glycophorin A/TER119 receptor which sorts to reticulocytes (26). CD71^+^ erythrocytes are rare in peripheral blood (27). Accordingly, mMEs express fewer cell-surface receptors than erythroblasts.

Since viruses and bacteria can replicate in a very short time relative to ME life span (>20 days in chicken, 120 days in human, 4–6 months in fish, and >500 days in turtles), life span of ME cannot explain erythrocyte resistance to infections. On the contrary, the absence of nuclear activity (due to enucleation in mammals and inactivation in other vertebrates) offers a likely explanation.

Enucleated cells have the advantage of reduced cell-surface receptors, allowing erythrocytes to evade microbial attachment, colonization, and invasion.

#### Parasitic Disease and Erythrocytes

About 130 *Plasmodium* species have been described in mammals, birds, and reptiles (28). Different species of *Plasmodium* can cause diseases in different hosts. In human, five species have been identified (*Plasmodium vivax, P. ovale, P. falciparum, P. malariae*, and *P. knowlesi*). Several species are found in chimpanzees *P. reichenowi, P. gaboni, P. falciparum*, and *P. gaboni*; in reptiles *P. mexicanum* and *P. floridense;* and *P. relictum* and *P. juxtanucleare* in birds (29).

*Plasmodium vivax* can invade bone marrow CD71^+^ reticulocytes (27). Experiments with erythrocytes from mice deficient in pyruvate kinase and expressing high levels of CD71 receptor show increased susceptibility to *P. yoelii* 17x-GFP (30). Flow cytometry shows that only 0.013% of mouse erythrocytes are parasitized by *P. chabaudi adami*. This number is reduced by 35% after protease treatment of erythrocytes for 30 min (31). Peripheral blood enriched for reticulocytes shows significant (2.2-fold) increase in *P. knowlesi* infection relative to normal blood (32, 33). Accordingly, only a small subset of red cells expressing a specific (unknown) receptor is susceptible to infection by malarial merozoites. This example of parasitism of an erythrocyte developmental stage(s) but not MEs is rare, it reflects the efficient role of epimmunity in protecting vertebrate MEs against malaria and other microbes.

#### Bacterial Diseases

Among pathogenic bacteria, some species are obligate or facultative intracellular parasites; *Legionella, Chlamydia, Anaplasma, Ehrlichia, Rickettsia, Coxiella, Brucella* spp., *Listeria monocytogenes, Erysipelothrix rhusiopathiae, Tropheryma whipplei, Shigella, Yersinia pestis, Francisella tularensis, Burkholderia pseudomallei, Burkholderia cenocepacia, Salmonella typhimurium, Edwardsiella tarda*, and *Mycobacterium* spp. (34, 35).

Experimentally Mehock et al. (36) showed that only 1% of feline red cells are infected *in vitro* by *Bartonella henselae*. Mändle et al. (37) have shown that *B. henselae* does not adhere, invade, nor infect human erythrocytes, yet it can invade and persist in CD34^+^ hematopoietic progenitor cells (HPCs). *B. quintana* most likely colonizes the bone marrow. It evades immune clearance and causes persistent and relapsing infections. Quiescent HPCs are resistant to *in vitro* infection with *Listeria monocytogenes, Salmonella enterica*, and *Y. enterocolitica* (37).

*Anaplasma marginale* (Rickettsiales: Anaplasmataceae) causes persistent infections in cattle erythrocytes and tick vectors (38). Anaplasmataceae species of the genera *Aegyptianella, Bertarellia, Cytamoeba, Eperythrozoon*, and *Haemobartonella* can infect erythrocytes and form cytoplasmic inclusions without any pathological consequences to host or erythrocytes (15). Possible explanations to the lack of bacterial pathogenic consequences may reside in the inability of red cells to uptake transferrin, iron, and other nutrients required for normal bacterial growth within red cells. Alternatively, the inability of bacteria to thrive inside red cells may be related to mitochondrial loss of function and activity in aging red cells, which may have similar impact on bacteria inside erythrocytes. A third possibility is that only a small subset of erythrocytes is susceptible to infections by Anaplasmataceae species. Other possibilities include lactic acid formation resulting from glycolysis as shown for cattle erythrocytes (39) and formation of reactive oxygen species (1, 40). All of these possibilities share a common denominator, that is, intracellular epimmunity.

Accordingly, MEs appear to resist infection and if infected appear to offer little, if any, support to the intracellular microbe.

Relevant to this issue is the interesting results reported by Wynn et al. Provision of immunosuppressive CD71^+^ (enucleated) reticulocytes to murine neonates before polymicrobial (bacterial) sepsis challenge did not affect animal survival. On the contrary, reduction of CD71^+^ after anti-CD71 treatment enhanced bacterial clearance (41). It is not clear if CD71^+^ cells harbored intracellular bacteria; since the depletion of these cells may have denied intracellular bacteria their sanctuary leading to their enhanced clearance in murine neonates.

Experimental enucleation using centrifugation in the presence of cytochalasin B generating enucleated cells known as cytoplasts may contribute significantly to our understanding of host–pathogen interaction. Cytoplasts retain several of cell activities including viral replication and protein synthesis (42). As shown by Yamamoto et al. (43), *Shigella flexneri* invades and multiplies within cytoplasts. Although, it was not clear from their work how they distinguished between intracellular and surface-bound bacterial cells despite the availability of simple techniques. Speert and Gordon distinguished between macrophage surface-bound and ingested *Pseudomonas aeruginosa* using lysozyme treatment (44).

#### Viral Interaction with Erythrocytes

Cells harboring active or partially active nuclei such as neurons, hepatocytes, B-cells, T-cells, and myocytes (45) support viral infections pending the expression of specific cell receptors of the corresponding viral ligand.

Viruses are obligate intracellular parasites; they depend on host cell for the provision of ATP, ribosomes, and aminoacyl-tRNAs. Most DNA and some RNA viruses also require nuclear functions for transcription and replication. Most of these requirements are not available in MEs.

##### Viral Attachment, Agglutination, Hemadsorption, and Cell Fusion

Receptor-mediated infections determine tissue tropism of viruses. The specific interaction between viruses and cell receptors is illustrated by the ability of viruses to agglutinate certain animal erythrocytes but not others (Table 1). Similarly, hemadsorption of chicken erythrocytes to mumps-induced syncytia of HeLa cell indicates selective mumps–erythrocyte interaction (46). Although several viruses are known to agglutinate or adsorb to erythrocytes, no viral replication has been reported to take place within vertebrate erythrocytes including Newcastle disease virus (NDV), mumps, or influenza B (2). PRCA is a genetic disease marked by the absence or reduction of nucleated red cells from the bone marrow. A similar transient form of the disease is caused by B19 infections (3, 47), which is a human parvovirus that infects and replicates in erythroid progenitor cells but adsorbs to the blood group P antigen (48). A later study (49) showed that human subjects who are deficient in P antigen are naturally resistant to B19 infections. Recombinant construct of B19 showed that P antigen alone which is distributed on several cell types and cell lines is insufficient to mediate internalization of the recombinant virus (50).

**Table 1:** Examples of erythrocytes agglutinated by different viruses.

Erythrocyte source	Agglutinating virus (species)	Reference
Chicken	Influenza	Tamm (2)
Human O-group erythrocytes	Influenza (H1N1)	Tsukasa et al. (51)
Human, goose, chicken, guinea pig, horse (poor agglutination)	Influenza (H5N1)	Louisirirotchanakul et al. (52)
African primate, gray monkey	Adenovirus (3,11,16,21), reoviruses, enteroviruses	Mutanda and Munube (53)
Red-tail monkey	Echovirus (7, 12)	Mutanda and Munube (53)
Albino rat	Adenovirus (10, 24, 27)	Mutanda and Munube (53)
Gray monkey and Albino rat	Reovirus (1,2)	Mutanda and Munube (53)
Sheep	Human cytomegalovirus	Bernstein and Stewart (54)

*In vitro*, expanded erythroid progenitor cells are easily infected with B19V, yet the virus is poorly produced from these cells. Viral productivity increases by ten folds when erythroid progenitor cells are incubated under hypoxic environment (1% oxygen) (1), these results suggested that intracellular reactive oxygen radicals contributed to controlling viral propagation. They concluded that the virus B19 sustains progenitor erythroid cells conductive to cell-surface–nuclear signaling system that involves transcription factors STAT5A and MEK/ERK pathways. B19 interaction with enucleated ME is diminished to near-harmless levels (1).

Agglutinating viruses elute spontaneously from erythrocytes, possibly by an enzymatic alteration of erythrocyte surface receptors (mucoproteins) (2). A possible conclusion is that agglutinating viruses, especially naked viruses are merely adsorbed to cells; they do not penetrate or fuse agglutinated erythrocytes as supported by the agglutination tests and hemadsorption photos. No reports of giant red cells. These agglutinating viruses do not cause disease in source animals; cytomegalovirus of human is not known to cause disease in sheep erythrocytes (Table 1). Fusogenic viruses require the ligation of specific receptors for fusion to take place. Ligation to surface structures does not necessitate fusion, internalization, or uncoating of the viruses. Simple experiment showing intact viruses after agglutination or uncoated nucleic acids can be accomplished by experiments duplicating Hershey–Chase experiment (55).

Cell cultures used to isolate viruses have to express the correct receptor; no single cell culture is capable of supporting all human viruses due to high specificity of attachment and infection. If we were made of a single cell type, we would have been destroyed by a single infection. Our tissue variations guarantee that one type of virus is incapable of assaulting all tissues or all developmental stages of a single cell as shown for B19 virus, many other viruses, and *P. vivax* (see Viral Interaction with Erythrocytes and Parasitic Disease and Erythrocytes, respectively).

##### Lysis of Red Cells

Snake venom effects include neurotoxicity, complement fixation, and induction of hemolysis (56), the paramyxovirus mumps was shown to lyse chicken, sheep, and Group-O human erythrocytes (57). New castle chicken disease virus (NDV) is known to lyse avian erythrocytes. *In vitro* experimental hemolysis of rooster erythrocytes by NDV ranges between 24 and 59% (58, 59).

Naïve chicken allantoic fluid shows significant inhibition of erythrocyte lysis. Allantoic fluid obtained from NDV challenged chicken embryos, significantly reduces NDV ability to lyse rooster erythrocytes (even after boiling) relative to naïve allantoic fluid of chicken embryos (60). Hemolysis of erythrocytes was not implicated in the death of any of the adult chicken killed by NDV experimental infections (45). Death rate of NDV oronasally infected chicken is 56%; dead chicken showed hemorrhage and other pathological signs (61). In another study, lethality was 58.30% for chicken, but only 2.94% for ducks infected with the same NDV in the same experiment (62). The mechanism by which NDV lyses avian erythrocytes remains unknown (59), especially since NDV does not replicate within avian erythrocytes (2). The fusion glycoproteins of NDV and other paramyxoviruses belong to the class I fusion protein group (63). Experimentally, Sendai virus (HVJ) fusion proteins are used to lyse cells, fuse cells, and fuse HVJ containing liposomes to erythrocytes (64), suggestive of a possible role of the fusion protein in cell lysis.

Adult chicken infected with NDV viscerotropic or neurotropic pathotype usually suffer secondary infections (45) indicating that NDV infections may not be lethal *per se*. The fact that some birds had survived the challenge is indicative of viral inability to kill all challenged birds, which may or may not have suffered a secondary bacterial infection.

##### Penetration and Replication

If a virus succeeded in penetrating an erythrocyte it will be trapped and unable to complete its cycle of replication due to unavailable resources to support viral replication.

However, inside non-mammalian erythrocytes, viral DNA may integrate into host chromosome(s), in doing so, there are two possibilities: the integration represents a dead end to the viral DNA where it will be degraded as erythrocytes die and are cleared by the phagocytic system. The second is a possible activation of avian nucleus and the erythrocyte. Such activation will be significant if it results in erythrocyte division or neoplasm formation.

These possibilities are eliminated from mammalian erythrocytes due to the complete absence of nucleus. Although nuclear inactivation can be viewed as an extreme case of epigenetic control, enucleation of mammalian erythroblasts represents the ultimate epigenetic control. Accordingly, enucleation can be viewed as a higher evolutionary level over nuclear inactivation.

#### Fungal and Yeast Infections

Intracellular infections by yeast appear to be limited to phagocytic cells; macrophages and neutrophils. Certain yeasts have developed mechanisms of immune evasion and survival against cytokines and lysosomal destruction. Latent and recurrent intracellular yeast infection caused by *Candida albicans* (65–67). *Histoplasma capsulatum* and *Cryptococcus neoformans* are found in macrophages and neutrophils (68, 69). *C. glabrata* (facultative intracellular parasite) survives and replicates inside macrophages as supported by direct microscopic evidence (70). Scientific reports concerning yeast or fungal association or infection of erythrocytes were not found.

## Physiological and Immunological Compatibility of Erythrocytes

Mature erythrocytes cannot perform several functions and must be compatible with other cells and tissues.

### Erythrocyte Deformability and Innate Immunity

In addition to their small size, ME must be flexible (deformable) in order to pass through the narrow network of capillaries (human capillaries are 3–10 µm in diameter) (12, 71). Enucleation of mammalian erythroblasts contributes to erythrocyte deformability and efficiency of oxygen-carrying capacity (26, 72, 73). Several lines of evidence show that loss of deformability results from ligating erythrocyte receptors such as complement receptor (CR1) by complement-opsonized microbes or other immune complexes. The loss of deformability plays an important role in the clearance of rigid red cells. Complement–erythrocyte interaction leads to the phosphorylation of the cytoskeletal (band 3) protein which limits erythrocyte deformability (74, 75). Rigidity of erythrocyte in cases of stomatocytosis, discocytosis, and antibody opsonization signals their removal by the phagocytic cells (76). In cases of bacterial infections, complement opsonized bacteria are captured by erythrocytes CR1 where erythrocytes become rigid causing slow microcirculation in the area of infection, eventually entering the liver and spleen where they interact with resident sinusoidal macrophages. They deliver captured bacteria and immune complexes to macrophage and carry on back to circulation (40, 74, 77).

*Plasmodium falciparum* evades immune clearance by maintaining gametocyte-infected red cells normally deformable; infected cells do not lose their deformability and remain in the circulation for a longer period. The drug sildenafil (viagra) induces rigidity of gametocyte-infected red cells due to protein kinase A-mediated phosphorylation leading to their removal. The phosphorylation of C-terminus serine residue of the protein SubTElomeric Variable Open Reading frame is a signal conveyed to the cytoskeleton through ankyrin, a complex that regulates red cell deformability (78). Erythrocyte deformability is subject to several factors; increased intracellular calcium ions result in rigid cells (74).

Relevant to complement–erythrocyte interaction is the human collectins, mannan-binding lectin, and surfactant protein A and D, which in the presence of an infectious agent may activate the lectin pathway (79).

### Erythrocytes Cannot Divide

Human orthochromatic erythroblasts lose the ability to divide as they exit the cell cycle (26). Human reticulocytes undergo several changes within 1–2 days after being released into the circulation, they mature into erythrocytes which lose an average of 15% of their hemoglobin over their lifespan. *In vitro*, reticulocytes are larger than erythrocytes and contain more RNA and hemoglobin relative to MEs (80). The number of erythrocytes is monitored and regulated by erythropoietin in response to levels of blood oxygen (81).

If hypothetical erythrocytes (HEs) had metabolically active nuclei and were capable of cell division, then new levels of homeostasis have to be attained by other organ systems. The immune system must handle excessive lysis, infections, and malignancies of HEs. Other systems also must reach homeostasis in response to HE activities. However, if HEs double their number by cell division, an increase in blood volume may lead to hypertension, increased blood viscosity, sluggish circulation, and stressed heart.

A regulatory feedback system coordinating HE numbers will be required; such a system must signal every HE to halt or commence cell division, a process that requires one or more receptors which in turn may become targets for microbial attack.

### Erythrocytes Cannot Be Sticky

In order to flow smoothly in the circulation without interfering with physiological functions of other cells, erythrocyte cell surface receptors and structures must be limited to a few permissible types that coat erythrocyte surface such as the highly glycosylated glycophorins. Such simple structure (72) should allow for a smooth erythrocyte (blood) flow. Blood transfusion between compatible human individuals is indicative of the simple basic surface structures expressed by human erythrocytes; unlike other tissue grafts and implants, which require histocompatibility matching. Scanning electron microscopy shows smooth erythrocyte surfaces relative to surfaces of activated platelets and leukocytes (72, 82). The simple surface structure reduces erythrocyte adherence to endothelia, leukocytes, bone marrow, cardiac muscle, cardiac valves, other erythrocytes, and microbes. Meanwhile, confinement of erythrocytes to the closed circulatory compartment prohibits its contact with other tissues (e.g., alveolar parenchyma, hepatocytes, fibroblasts, and neurons).

### Erythrocytes Cannot Perform Extravasation (Diapedesis)

As leukocytes respond to cytokines and signals of inflammation, they extravasate (exit the circulation) to other tissues. Since the main function of erythrocytes is in gas exchange, it is counterproductive for erythrocytes to wonder outside the circulation, they do not perform extravasation and remain trapped inside the circulation, thereby defining the closed circulatory system. Inability of red cells to respond to extravasation signals indicates the importance of enucleation (or nuclear inactivation) in the prevention of extravasation and cell division (see Other Issues Resolved by Enucleation or Nuclear Inactivation).

### Erythrocytes Must Not Interact Non-Specifically with Serum Molecules

Signaling molecules are released in the blood at very low concentration; if MEs were to capture or non-specifically interfere with such signaling molecules, the signal will be attenuated or interrupted before reaching its target receptor. MEs are depleted from transferrin CD71 receptor (26) since they do not require transferrin, accordingly they do not compete with other cells for signals or nutrients. MEs transport glucose *via* GLUT1, which has low affinity to glucose, they also utilize fructose *via* GLUT5 (83). GLUT 5 has been reported on enterocyte, muscle, kidney, brain, and testis cells, but has not been reported on leukocytes.

## Other Issues Resolved by Enucleation or Nuclear Inactivation

### Intracellular Hemoglobin vs Extracellular Globin of Lower Life Forms

In unicellular organisms and simple organisms (e.g., sponges), O_2_/CO_2_ exchange with the environment is direct. In arthropods including insects, oxygen transport is carried out by extracellular globins; hemerythrins and hemocyanins (84–86). Backswimmer insects (*Anisops* and *Buenoa*) hemoglobin is located intracellularly in the cells of large abdominal trachea (87). Vertebrates adopted an intracellular hemoglobin system (the red cells, erythrocytes, or RBCs) for gas exchange. This intracellular system has overcome several problems associated with extracellular globins; serum viscosity and the short half-life of extracellular globins relative to the long half-life of intracellular hemoglobin (88–90). Since vertebrates have closed circulatory systems, a third problem was resolved by confining hemoglobin and erythrocytes to the circulatory system and preventing them from leaking outside the circulation. The importance of confining hemoglobin to the closed circulation is emphasized by the plasma haptoglobin that binds serum hemoglobin and prevents it from causing renal damage (91). Excess soluble hemoglobin can deplete haptoglobin which appears diagnostically in urine (92). Leakage of hemoglobin outside the circulation is counterproductive. Whereas blood flow rate is about one cycle per minute (93), leakage of hemoglobin or ME outside the circulation will prolong the flow rate far beyond the 1 min rate. Therefore, localization of hemoglobin intracellularly is a suitable solution for closed circulatory systems. Leukocytes exit circulation *via* extravasation (diapedesis) (94), whereas erythrocytes are incapable of extravasation, they remain to define the closed circulation of vertebrates. While the intracellular entrapment of hemoglobin has solved viscosity, globin turnover, and hemoglobin leakage problems on one hand, it created significant problems on the other. In addition to epimmunity and deformability, three other problems that erythrocytes must contend with are discussed in the following sections.

### High Erythrocyte Surface Area to Volume Ratio for Efficient Gas Exchange

The absence of nucleus from mMEs allows the cell to acquire the biconcave-discoid shape, hence, creating a high A/V ratio. Human biconcave RBCs have a high ratio: ~1.53/μm, whereas the ratio for the spherical shape, i.e., nucleated RBC (erythroblast) is ~0.833/μm (95).

Non-mammalian vertebrates (birds, fish, amphibians, and reptiles) possess nucleated erythrocytes that contain cytoplasmic organelles (96) and functional mitochondria (14). Avian chromatin condensation, nuclear collapse, and inactivation allow avian erythrocytes to appear as flat, thin, flexible, elliptic cells under scanning electron microscope ~(2.24 × 7 µm wide) (97) with a predicted A/V ratio of ~1.53/μm (97–100).

### Erythrocytes Must Maintain a Continuous Supply of Oxygen to All Cells

Uninterrupted supply of oxygen is maintained by the large numbers of circulating erythrocytes (2–3.1 × 10^13^ in adult human), timely heartbeat, and normal breathing rate (at rest human breathing rate is 12–16 times per minute) (101). The size and number of circulating human erythrocytes are subject to variation; oxygen concentration, gender, and alleles of *TRIM58* gene resulting from SNPs (5).

## Comments and Recommendations

The “Epimmunity theory” predicts that no single disease should equally affect members of a given vertebrate species. Epimmunity is clearly utilized by free-living and parasitic unicellular organisms including bacteria which may resist bacteriophages and antibiotics. Accordingly, zygotes of multicellular organisms and their eventual developmental stages must be epimmune. Published research in different biological areas supports the existence of single cell defenses (epimmunity). Similar to other biological systems, epimmunity is subject to breakdown, abnormality, and failure as well.

Genetically, cells safeguard their critical metabolic pathways by having multiple alternative pathways such as the intricate regulation of checkpoint/cyclins/kinases controlling the cell cycle (102), repair mutations, and can circumvent mutations in many cases by alternative splicing of pre-mRNA (103), by utilizing an alternative metabolic pathway, and by suppressing mutations. In diploid animal cells, recessive mutations are suppressed by the presence of a functional allele.

Epimmunity should be incorporated as a genuine branch of immunology since it is not accounted for in any field of knowledge. Epimmunity cannot be replaced or compensated by any other component including those of the immune system.

Immune tolerance and autoimmunity should be reevaluated from the epimmunity point of view; tolerance breakdown or autoimmunity may actually result from “epimmunity breakdown.” Since it takes at least two entities to interact in an autoimmune disease, either entity can be defective. Therefore, in an autoimmune disease, the breakdown could have afflicted the immune cell or its target cell (antigen). Epimmune cells will become susceptible to certain diseases upon breakdown in their epimmunity.

Understanding the properties of erythrocytes in relation to diseases and cancer biology may open new avenues toward nuclear inactivation in cancer research. Inhibition of extravasation process in metastatic cancer and leukemia may prevent their metastasis, especially prior to medical or surgical intervention.

Having inactive nucleus carries a risk of reactivation; reentering cell cycle or gene expression as a result of external stimuli such as exposure to radiation, chemical agents, or biological agents. This area of gene and nuclear reactivation can be experimentally exploited to understand the mechanisms responsible for inactivation/reactivation as attested by earlier studies (13, 18, 22).

I propose targeting extravasation of metastatic cancer for inhibition by transient, reversible, and partial (or complete) nuclear inactivation as an important strategy in combating metastasis and cancer cell proliferation. Applying miRNA to inhibit transcription of cellular deformability genes (104), which should not affect erythrocyte deformability since it is enzymatically regulated; Section “Erythrocytes Deformability and Innate Immunity.” Extravasation may be inhibited by targeting cytoplasm streaming with drugs such as cytochalasin B that affect animal cell microfilaments (105); injecting the drug into the arteriole that feeds into a tumor may prove valuable in controlling or preventing metastasis.

Other areas of importance include *ex vivo* erythrocytes loading with therapeutic drugs for programmed drug delivery (106) or as proposed by PEGylation system (107).

## Author Contributions

The author confirms being the sole contributor of this work and approved it for publication.

## Conflict of Interest Statement

The author declares that the research was conducted in the absence of any commercial or financial relationships that could be construed as a potential conflict of interest.
